# The Role of Novel Antifungals in the Management of Candidiasis: A Clinical Perspective

**DOI:** 10.1007/s11046-023-00759-5

**Published:** 2023-07-20

**Authors:** Eloy E. Ordaya, Josh Clement, Paschalis Vergidis

**Affiliations:** 1 Division of Public Health, Infectious Disease, and Occupational Medicine, Mayo Clinic, 200 First St SW, Rochester, MN 55905 USA; 2 Department of Pharmacy, Mayo Clinic, Rochester, MN USA

**Keywords:** Candidemia, Candidiasis, Rezafungin, Ibrexafungerp, Fosmanogepix, Oteseconazole

## Abstract

Mucosal and invasive candidiasis can be challenging to treat in the setting of drug intolerance, antifungal resistance, drug–drug interactions, or host immune status. Antifungals with novel mechanisms of action and distinct pharmacokinetic/pharmacodynamic properties have been developed in recent years. Rezafungin is an echinocandin with high-tissue penetration and an extended half-life that allows for once-weekly administration, making it a convenient treatment option for invasive candidiasis while obviating the need for central catheter placement. Ibrexafungerp is an oral glucan synthase inhibitor that is active against most echinocandin-resistant *Candida* species. At present, it is approved for the treatment of acute vulvovaginal candidiasis and is under investigation as an oral step-down therapy following initial treatment with an echinocandin for cases of invasive candidiasis. Oteseconazole is a long-acting tetrazole that exhibits a higher affinity for the fungal enzyme *CYP51*, resulting in a potentially lower risk of drug–drug interactions and side effects compared to other azoles. It is currently approved for the treatment of recurrent vulvovaginal candidiasis. Fosmanogepix has a novel mechanism of action and potent activity against several *Candida* strains resistant to other antifungals. Due to its considerable bioavailability and tissue penetration, it holds promise as a potential treatment option in patients with invasive candidiasis, including those with chorioretinitis or meningitis. Results from clinical trials and observational studies will further delineate the role of these agents in the management of candidiasis. As the usage of these novel antifungals becomes widespread, we expect to acquire a greater understanding of their efficacy and potential benefits.

## Introduction

The genus *Candida* comprises more than 200 species, many belonging to the human microbiota of the skin, gastrointestinal tract, and vaginal flora [1–3]. *Candida* species can cause a wide range of infections, from localized mucosal disease (e.g., vulvovaginal candidiasis) to deep-seated invasive infection and candidemia [2, 4]. Approximately 90% of infections are caused by *Candida albicans*, *Nakaseomyces glabrata* (formerly *Candida glabrata*), *Candida parapsilosis*, *Candida tropicalis*, and *Pichia kudriavzevii* (formerly *Candida krusei*) [2, 5]. *C. albicans* remains the most frequent species causing candidiasis. However, the prevalence of non-*albicans Candida* species infection has steadily increased in recent years [6, 7]. Compared to *C. albicans* isolates which commonly remain susceptible to fluconazole, non-*albicans* species demonstrate variable susceptibility to antifungal agents [7–9]. Furthermore, *Candida auris* has emerged as a multidrug-resistant species that can be associated with outbreaks in healthcare settings [3, 10, 11].

Established antifungal agents for managing candidiasis belong to four drug classes: azoles, polyenes, echinocandins, and pyrimidine analogs (flucytosine). Azoles and polyenes act at the level of the fungal membrane, echinocandins on the fungal cell wall, and flucytosine impairs nucleic acid synthesis [8, 9]. Antifungal treatment selection is based on multiple factors, including the host immune status, the extent of infection, prior drug tolerance, and antifungal resistance [12]. Antifungal resistance can be intrinsic (e.g., fluconazole-resistant *P. kudriavzevii*) or acquired (e.g., echinocandin-resistant *N. glabrata*), with the latter typically occurring following prolonged antifungal exposure [7–9]. Resistance mechanisms include the alteration of the binding sites through the enzyme-encoding gene *ERG11* mutation and overexpression of the efflux pumps *CDR1, CDR2,* or *MDR1* (for azoles), and amino acid substitutions in the *FKS* subunits of the glucan synthase (for echinocandins) [12–15]. *Candida* biofilm formation is potentially contributing to the emergence of resistance, given the decreased ability of antifungal agents to penetrate biofilms and reach the intended site of action [9, 12, 13].

Novel antifungals with activity against *Candida* species have been developed in recent years. Herein, we review the available data from pre-clinical and clinical studies on rezafungin, ibrexafungerp, oteseconazole, and fosmanogepix. Although real-world data is currently lacking, we provide examples of challenging cases and discuss the potential role of these novel antifungal agents.

## Rezafungin

### Case 1

A 43-year-old man with end-stage renal disease receiving intermittent hemodialysis via a tunneled central venous catheter was admitted for fever. The patient was started on empiric antibiotic treatment with cefepime and vancomycin. Blood cultures collected from the hemodialysis catheter and peripheral blood grew fluconazole-resistant *C. auris.* The minimum inhibitory concentration (MIC) of fluconazole was 128 μg/mL, micafungin MIC was 4 μg/mL, and rezafungin MIC was 0.25 μg/mL (susceptible if MIC ≤ 0.5 μg/mL [16]). Echocardiogram showed no valvular insufficiency or vegetations. The fundoscopic exam was unremarkable. The hemodialysis catheter was removed. Rezafungin was considered an appropriate treatment option for this patient and was administered via a peripheral catheter in 2 doses (day 1, day 8).

Rezafungin (formerly CD101) is a novel echinocandin derived from anidulafungin with potent in vitro and in vivo activity against *Candida*, *Aspergillus,* and *Pneumocystis* [9, 17]. Rezafungin has poor activity against *Cryptococcus* species and rare mold, such as Mucorales, *Fusarium*, and *Scedosporium* [18]. Rezafungin demonstrates activity against azole-resistant *Candida* spp (including *N. glabrata* and *C. auris*). Similar to other echinocandins, *FKS* mutations lead to increased rezafungin MICs. However, a once-weekly dosing regimen achieved ≥ 90% probability of target attainment associated with effective drug target exposures [19]. Due to a chemical modification that reduces degradation, rezafungin has improved tissue penetration compared to other echinocandins and prolonged half-life (133 h in humans), allowing once-weekly dosing [20–22]. In addition, rezafungin has potent activity against *Candida* and *Pneumocystis* biofilms [23, 24]. In a murine model, rezafungin achieved faster and higher concentration in hepatic tissue and had a more uniform distribution in necrotic lesions compared to micafungin [25, 26]. Rezafungin was compared to caspofungin in the phase 2 STRIVE trial. The higher front-loaded exposure (400 mg loading dose followed by 200 mg weekly dose) correlated with mycological eradication at day five and day fourteen compared to caspofungin [27]. The phase 3 ReSTORE trial demonstrated non-inferiority of rezafungin compared to caspofungin for the primary endpoints of global cure (clinical, radiological, and mycology eradication) and 30-day mortality in patients with candidemia and/or invasive candidiasis [28]. Despite the higher front-loaded exposure, the safety and tolerability of rezafungin have been reported to be similar to other echinocandins [27, 29]. Rezafungin is stable in hepatocytes with no biotransformation indicating a low potential for drug–drug interactions [30]. Rezafungin is mainly excreted in feces (< 1% excreted unchanged in urine) [30]. Dose adjustment is not required for patients with renal or hepatic dysfunction [31].

## Comment

Echinocandins are preferred over azoles for the initial treatment of candidemia. In two randomized clinical trials, echinocandins demonstrated superior efficacy compared to azole antifungals [32, 33]. Similar conclusions were drawn from observational studies and led to the guideline recommendation of initiating treatment with an echinocandin and transitioning to an oral azole after clinical stability has been achieved [34]. By extrapolating from the existing evidence, we anticipate that rezafungin will have superior efficacy to fluconazole for the initial management of candidemia. In phase 2 and phase 3 clinical trials comparing rezafungin to caspofungin, eligible participants could have received standard-of-care antifungal therapy with an approved echinocandin prior to enrollment (but for no longer than 48 h) [27, 28]. It is known that timely initiation of antifungal treatment can affect clinical outcomes. Faster blood culture clearance was observed in patients receiving rezafungin compared to caspofungin. Once rezafungin is used in clinical practice, we will assess wheter this finding correlates with superior clinical outcomes.

*Nakaseomyces glabrata* exhibits reduced susceptibility to fluconazole (isolates are susceptible dose-dependent or resistant). *Pichia kudriavzevii* demonstrates inherent resistance to fluconazole. For bloodstream infections caused by fluconazole-resistant *Candida* species, our practice is to administer an echinocandin for the duration of treatment without stepping down to an oral azole. Given its prolonged half-life, rezafungin is conveniently dosed once weekly and will likely be favored over other echinocandins in this setting (as exemplified by case 1 in this review).

Based on the activity of other echinocandins, we anticipate that rezafungin could potentially be used to treat *Candida* endocarditis. Rezafungin has demonstrated potent activity against *Candida* biofilms [23] which can be formed in native and prosthetic valves. People who inject drugs are at high risk for *Candida* endocarditis, and weekly antifungal dosing sparing central catheter placement may be the preferred approach in this patient population. We note, however, that patients with infective endocarditis were excluded from the STRIVE and ReSTORE trials. Given its activity against biofilm, rezafungin may also be used in the treatment of vascular graft infections. Similarly, it may be used to treat candidemia in patients with left ventricular assist devices or those receiving extracorporeal membrane oxygenation support.

Drug penetration and distribution within infected tissue are determinants of clinical response. In a murine model of intra-abdominal candidiasis, rezafungin accumulated faster and persisted longer in hepatic tissue compared to micafungin [25]. Importantly, rezafungin demonstrated a balanced distribution within necrotic lesions, whereas micafungin provided a higher signal in the outer rim compared to the necrotic center. These properties may be particularly beneficial in the management of infections with high fungal burden and the presence of necrotic tissue (i.e., multiple intraperitoneal or hepatic abscesses, infected pancreatic necrosis). Primary *Candida* peritonitis occurs in the absence of an apparent breach of the gastrointestinal tract. The condition is typically encountered in patients with cirrhosis and has been associated with significant mortality compared to other forms of intra-abdominal candidiasis [35]. Future research will show whether rezafungin can improve the outcomes of primary peritonitis. We are also interested to see whether rezafungin will outperform other echinocandins in the treatment of hepatosplenic candidiasis, a deep-seated form of candidiasis among neutropenic patients.

Pharmacokinetic studies have shown that the concentration of echinocandins within the pleural fluid is lower compared to plasma [36, 37]. In a retrospective cohort study, patients with *Candida* pleural empyema treated with an echinocandin had higher 100-day mortality compared to those treated with fluconazole [38]. Given its pharmacokinetic properties, rezafungin may achieve higher concentrations in the pleural space resulting in superior clinical outcomes. Clinical studies on pleural fluid concentration will help to define its potential role for this indication. Similar to other echinocandins, rezafungin has poor penetration into the central nervous system and is not recommended for the treatment of *Candida* endophthalmitis or meningitis.

Echinocandin resistance mediated by *FKS* mutations can emerge after prolonged drug exposure. It has been postulated that intra-abdominal candidiasis (mainly in the form of abscesses) provides a reservoir for the emergence of resistance [39]. Given the front-loaded exposure and higher tissue penetration, rezafungin may be associated with a lower risk of emergence of resistance compared to the other echinocandins. Hence, rezafungin may become the preferred agent if prolonged antifungal treatment is planned; however, more clinical data on the safety of long-term use is needed. *Candida auris* has gained attention in recent years due to its potential for multidrug resistance and persistence in the environment leading to hospital outbreaks. In vitro studies have shown higher potency of rezafungin compared to the other echinocandins [40, 41]. It remains to be seen how rezafungin will perform compared to the other echinocandins in the treatment of *C. auris* infections in clinical practice.

In clinical trials, rezafungin demonstrated a similar safety profile to caspofungin with limited drug–drug interactions. In animal studies, unexpected tremors were observed. It is unclear whether neurologic side effects pose a safety concern in humans. Of note, patients with severe ataxia, tremor, or peripheral neuropathy were excluded from the clinical trials. We will learn more about the occurrence and magnitude of these potential side effects once rezafungin is regularly used.

## Ibrexafungerp

### Case 2

A 55-year-old man with a history of poorly controlled diabetes and multivessel coronary artery disease underwent coronary artery bypass graft surgery complicated by postoperative *N. glabrata* sternal osteomyelitis and received treatment with a prolonged course of caspofungin. The patient presented to the hospital complaining of persistent pain and recurrent drainage from the sternal incision. He underwent surgical debridement, and tissue cultures grew *N. glabrata* resistant to fluconazole (MIC 128 μg/mL) and caspofungin (MIC 1 μg/mL), and intermediately susceptible to anidulafungin (MIC 0.25 μg/mL) and micafungin (MIC 0.12 μg/mL). MIC to ibrexafungerp was 0.5 μg/mL (proposed epidemiological cut-off value for non-wild-type *N. glabrata* MIC > 1.0 μg/mL [42]). In this case, ibrexafungerp demonstrated in vitro activity against fluconazole- and echinocandin-resistant *N. glabrata*.

**Fig. 1:**
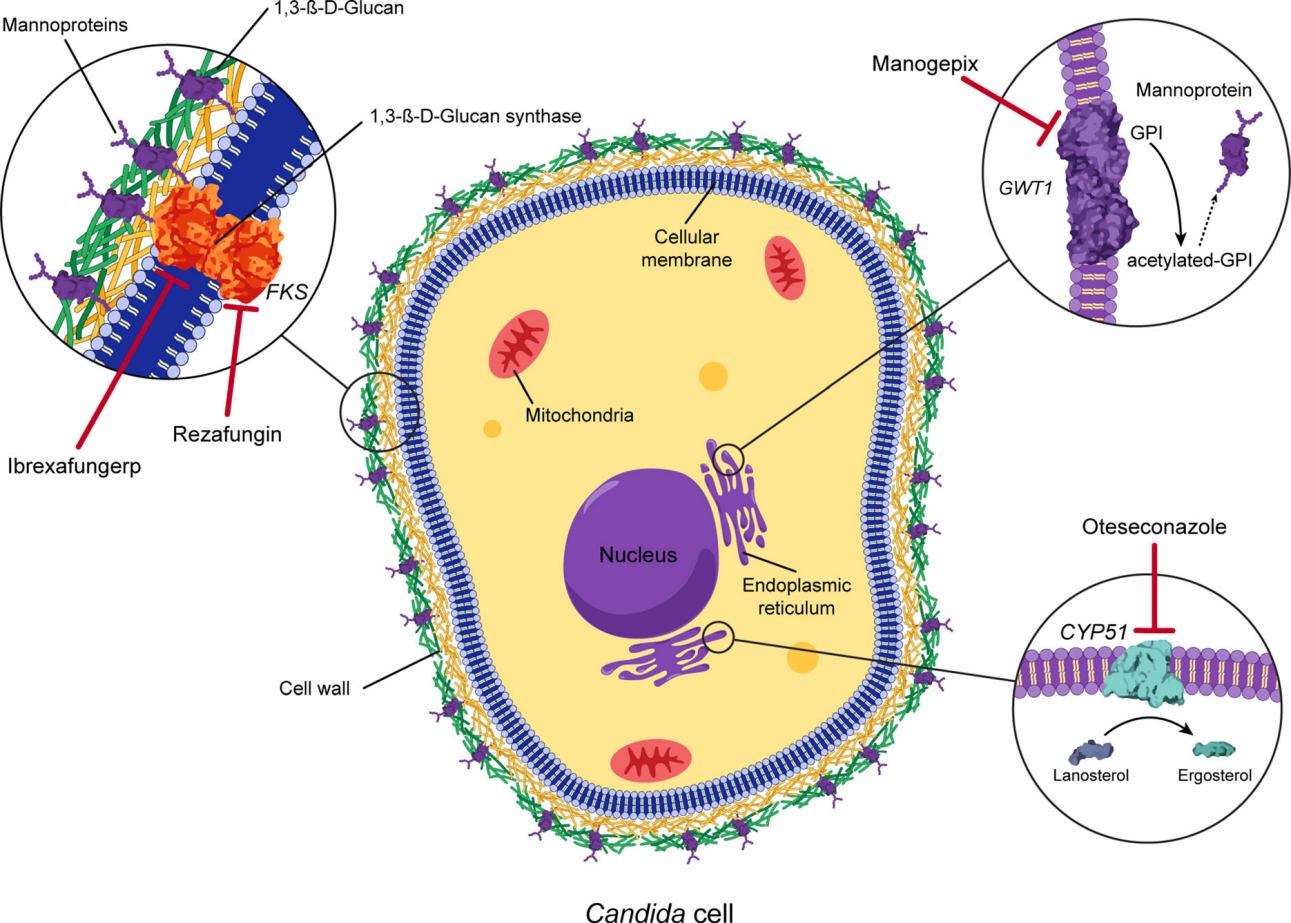
Mechanism of action of novel antifungals with activity against *Candida* species. Rezafungin and ibrexafungerp inhibit the cell-wall enzyme complex (1,3)‐β‐d‐glucan synthase at different subunits. *FKS* genes encode (1,3)‐β‐d‐glucan synthase. Manogepix, the active moiety of fosmanogepix, inhibits the fungal acetyltransferase enzyme (*Gwt1*) in the endoplasmic reticulum, blocking the acetylation of inositol and preventing the biosynthesis of glycosylphosphatidylinositol, thus, affecting the function of mannoproteins. Oteseconazole inhibits the fungal *CYP51* enzyme, blocking the conversion of lanosterol to ergosterol

Ibrexafungerp (formerly SCY-078) is an oral glucan synthase inhibitor and the first triterpenoid antifungal class member that has activity against *Candida*, *Aspergillus*, and dimorphic fungi [12, 43–46]. It lacks reliable activity against Mucorales and *Fusarium* [44, 45, 47]. Ibrexafungerp is a semi-synthetic derivative of enfumafungin and disrupts fungal cell wall synthesis by inhibiting (1,3)-β-d-glucan synthase, acting on the same target as echinocandins (Fig. 1). However, its distinct binding site to the glucan synthase only partially overlaps with the echinocandins. Hence, ibrexafungerp retains activity against most echinocandin-resistant *Candida* species [43, 46, 48–50]. Resistance to ibrexafungerp can occur in the presence of *FKS* mutations, especially with specific amino acid changes in the subunit *FKS2*. Activity is variable in the presence of *FKS* mutations, although it is still considered more potent than echinocandins [44]. Studies in *C. auris* have demonstrated potent antibiofilm activity and interruption of cell division [51, 52]. In contrast to the echinocandins, ibrexafungerp has the advantage of oral bioavailability with a prolonged half-life (30 h) and a larger volume of distribution with excellent tissue penetration in the liver, lung, kidney, spleen, skin, and bone [44, 47]. Of note, ibrexafungerp achieves reduced concentration in urine and has poor penetration into the central nervous system [46]. In an open-label trial of patients with invasive candidiasis initially treated with an echinocandin, step-down therapy to ibrexafungerp showed similar favorable response rates compared to standard-of-care treatment [43]. A phase 3 clinical trial aimed to evaluate the efficacy and safety of ibrexafungerp as a step-down therapy following caspofungin in patients with candidemia and invasive candidiasis is currently ongoing [53].

Ibrexafungerp has been studied in patients with a wide range of fungal infections that have been refractory to or intolerant of standard antifungal treatment [54]. As it targets an enzymatic pathway not found in humans, ibrexafungerp is well-tolerated. The most commonly reported side effects were abdominal pain, diarrhea, nausea, and vomiting [43, 46]. Ibrexafungerp undergoes extensive hepatic metabolism, with elimination mainly via feces and bile (∼ 90%) and minimally through urine (< 2%) [46, 47, 55]. No dosage adjustment is recommended in patients with renal dysfunction or mild-to-moderate hepatic impairment. It has not been studied in patients with severe liver dysfunction. Ibrexafungerp is contraindicated in pregnant patients, as fetal toxicity was observed in animal studies [56].

## Comment

Ibrexafungerp has been approved as a single-day oral treatment for uncomplicated vulvovaginal candidiasis (VVC). Ibrexafungerp is a treatment option for infection caused by fluconazole-resistant strains. In contrast to azoles, it retains activity in the low vaginal pH environment. We note, however, that patients infected with fluconazole-resistant *Candida albicans* isolates were not included in VANISH 303 [57] and VANISH 306 [58]. In these phase 3 clinical trials on VVC, ibrexafungerp was compared to placebo (and not to fluconazole).

Treatment of vaginitis caused by *N. glabrata* is challenging. Even for isolates that are in vitro susceptible to voriconazole, failure rates to azole therapy are high. Ibrexafungerp is a reasonable treatment option in this setting. We note that < 10% of infections were caused by non-*albicans Candida* strains in the clinical trials cited above. For the treatment of *P. kudriavzevii* vaginitis, we recommend the use of azole vaginal creams or suppositories (i.e., clotrimazole, miconazole, or terconazole), as data on the activity of ibrexafungerp against *P. kudriavzevii* is conflicting [42, 59].

In recurrent VVC, vaginal swab cultures should always be obtained for identification of *Candida* to the species level and azole antifungal susceptibility testing. We recommend treatment with ibrexafungerp for azole-resistant isolates. Future studies will better define the appropriate duration, but courses longer than single-day treatment may be required. In a phase 3 trial, patients with recurrent VVC were treated with fluconazole followed by monthly ibrexafungerp for 6 months [60]. More patients in the ibrexafungerp arm remained infection-free at the end of treatment compared to placebo (65.4% versus 53.1%). Based on these findings, ibrexafungerp was approved by the FDA for extended treatment. The efficacy of extended treatment for infection caused by fluconazole-resistant isolates will need to be studied further. Based on animal studies, ibrexafungerp may cause fetal harm, and use in pregnancy is unfortunately contraindicated similarly to azole therapy. Data collection on infant outcomes following exposure is ongoing.

The efficacy and safety of ibrexafungerp for the treatment of candidemia and invasive candidiasis are currently being studied in a phase 3 randomized clinical trial (MARIO) [53]. Eligible participants are initially treated with an intravenous echinocandin and are randomized to receive ibrexafungerp or fluconazole as step-down therapy. The primary outcome is 30-day mortality. If shown to be non-inferior to fluconazole, ibrexafungerp will be a treatment option for step-down therapy. We believe that it is crucial to understand the role of ibrexafungerp as step-down therapy for fluconazole-resistant *Candida* species (e.g., *N. glabrata*, *C. auris*). Transitioning from intravenous to oral therapy will facilitate the care of patients with invasive candidiasis.

Ibrexafungerp has been studied in the open-label FURI trial in patients who have been intolerant of standard antifungal treatment [54]. Intolerance to azoles is commonly related to hepatotoxicity, whereas allergic reactions are less common. Ibrexafungerp may be used as an alternative to azole therapy in patients with QT prolongation. Ibrexafungerp may be favored over itraconazole or voriconazole in patients with cirrhosis, although more clinical data is needed. Due to its unique binding characteristics to the glucan synthase, ibrexafungerp may retain activity against echinocandin-resistant *N. glabrata and C. auris*. The findings of the FURI trial will inform us of its role in refractory candidiasis.

Notably, ibrexafungerp achieves poor concentration in the cerebrospinal fluid, and the drug should not be used for the treatment of central nervous system infections (at least not as monotherapy). Treatment options for lower urinary tract infections caused by fluconazole-resistant *Candida* species are limited due to the poor urine concentration of other azoles and echinocandins. Ibrexafungerp undergoes extensive hepatic metabolism, and < 2% is recovered unchanged in the urine [55]. Given its tissue distribution, we believe that ibrexafungerp could be a treatment option for *Candida* pyelonephritis. Its role in cystitis will need to be elucidated in future studies. We also expect to learn more about its efficacy compared to the echinocandins in biofilm-associated infections such as endocarditis, osteomyelitis, and device infections.

## Oteseconazole

### Case 3

A 62-year-old woman with a history of poorly controlled diabetes and recurrent episodes of VVC caused by *P. kudriavzevii* was previously treated with clotrimazole vaginal cream. The patient returns complaining of pruritus and vaginal discharge. On physical exam, there are signs of severe vaginal inflammation. Vaginal swab culture grows *P. kudriavzevii*. Oteseconazole is indicated for this patient of non-childbearing age with recurrent VVC caused by a *Candida* species intrinsically resistant to fluconazole.

Oteseconazole (formerly VT-1161) is a novel oral tetrazole that inhibits the fungal *CYP51* enzyme lanosterol 14α-demethylase (Fig. 1) and has potent activity against *Candida* species (including *N. glabrata* and *P. kudriavzevii)*, *Cryptococcus* species, *Coccidioides immitis/posadasii*, and *Trichophyton* spp [44, 61–63]. Oteseconazole resistance mechanisms can vary but may be similar to triazole resistance conferred by *ERG11* mutations or overexpression of the efflux pumps *CDR1* and *MDR1* [44]. In contrast to other azoles containing an imidazole or triazole moiety that binds to the human cytochrome, oteseconazole has a tetrazole moiety with a greater specificity (2000-fold) for the fungal *CYP51* compared to human *CYP450* enzymes. Hence, oteseconazole is possibly associated with a lower risk for drug–drug interactions and adverse events [63–65]. Oteseconazole has an oral bioavailability of 40–70% and a prolonged long half-life of 138 days leading to sustained plasma levels. It has high tissue penetration, with studies showing comparable concentrations in vaginal tissue and blood [64, 65]. Oteseconazole does not undergo significant metabolism and is mainly excreted via feces and bile, with low levels recovered in urine [63]. No dose adjustment is recommended in patients with mild-to-moderate renal or hepatic impairment [66]. However, its use is not recommended in patients with severe renal or hepatic impairment due to the lack of safety information.

In a phase 3 clinical trial, oteseconazole was superior to placebo in preventing recurrent VVC [67]. Participants presenting with acute VVC entered an induction phase in which they were randomly assigned to receive oteseconazole or fluconazole (2:1). Cure rates were similar in both arms. Following the 2-week induction phase, participants with resolved VVC entered the maintenance phase and received oteseconazole or placebo weekly for 11 weeks. The recurrence rate through week 50 was 5.1% with oteseconazole compared to 42.2% with placebo (*p* < 0.001). In this study, 23.9% of *Candida* isolates at baseline were identified as non-*albicans* species. In 2022, the FDA approved the use of oteseconazole for women with a history of recurrent VVC. Based on animal studies demonstrating fetal harm, oteseconazole is contraindicated in pregnant and lactating women [66]. Oteseconazole has been tolerated well in clinical trials, with the most frequently reported adverse reactions being headache and nausea [67, 68].

## Comment

Recurrent VVC is a chronic debilitating condition that significantly affects the quality of life of millions of women worldwide [69]. Oteseconazole has been approved for recurrent VVC and has potential benefits over other azoles related to its long half-life, activity against fluconazole-resistant *Candida* species, lower risk for drug–drug interactions, and adverse events. As with other azoles, it is contraindicated in women that are pregnant, lactating, or of childbearing age. Given its pharmacological properties, oteseconazole could possibly have a role in other forms of mucosal or invasive candidiasis. However, it has only been studied in recurrent VVC.

## Fosmanogepix

Fosmanogepix (formerly APX001) is a guanosine monophosphate inhibitor with potent in vitro activity against *Candida*, *Cryptococcus*, *Aspergillus*, *Fusarium,* and *Scedosporium* spp [44, 70, 71]. It lacks activity against *P. kudriavzevii* and has variable activity against *Rhizopus*, *Lichtheimia,* and *Mucor* [71–74]. This first-in-class antifungal is the prodrug of manogepix, a molecule that inhibits the fungal acetyltransferase enzyme *Gwt1* in the endoplasmic reticulum (Fig. 1) [44, 75]. The inhibition of *Gwt1* affects the anchoring of mannoproteins to the fungal cell wall, impairing adherence to mucosal and epithelial surfaces, compromising the cell wall integrity, and affecting biofilm formation [74–76]. In vitro studies have shown that resistance to fosmanogepix can be acquired after drug exposure, primarily due to amino acid substitutions within *Gwt1* or overexpression of efflux pumps [44]. Fosmanogepix can be administered intravenously or enterally. It has a high oral bioavailability (> 90%) and achieves excellent concentrations in the eye and central nervous system in animal models [75, 77].

A phase 2 clinical trial demonstrated the safety and efficacy of fosmanogepix in patients with candidemia [78, 79]. Another clinical trial that aimed to evaluate the role of fosmanogepix in patients with candidemia/invasive candidiasis caused by *C. auris* was terminated early due to the impact of COVID-19 on trial-related activities [80]. In a planned phase 3 clinical trial, the safety and efficacy of fosmanogepix will be studied in patients with candidemia and invasive candidiasis [81]. Two-thirds of participants will receive intravenous fosmanogepix followed by an optional transition to oral formulation. One-third will receive standard care with caspofungin followed by transition to oral fluconazole.

The prolonged half-life of approximately 60 h allows once-daily dosing [75, 82]. In trials, fosmanogepix has been well tolerated, likely due to its fungal-specific activity, and was only associated with mild and transient adverse events (most commonly headache). It is primarily cleared by biliary/fecal excretion [75, 82]. Clinical trials have demonstrated that fosmanogepix is not associated with worsening renal function in patients with chronic kidney disease. An ongoing clinical trial is evaluating its safety in patients with hepatic dysfunction [83].

## Comment

Its novel mechanism of action allows fosmanogepix to retain potent activity against various resistant *Candida* strains, except *P. kudriavzevii*. Additionally, its high penetration in organs where other antifungals do not achieve adequate concentrations, including ocular tissue and central nervous system, makes fosmanogepix a potential treatment option in patients with intolerance or resistance to standard antifungal treatment. Given its high bioavailability and once-a-day dosing, fosmanogepix may be an option for step-down therapy in patients with invasive candidiasis, including *Candida* chorioretinitis or meningitis. Further studies are needed to evaluate its utility in this scenario.

## Conclusion

Both mucosal and invasive candidiasis can be challenging to treat in the setting of drug intolerance, antifungal resistance, drug–drug interactions, or host immune status. Despite the use of effective antifungal agents, candidemia continues to be associated with significant mortality. Fortunately, several novel antifungal agents are being studied and approved for clinical use. As we obtain additional clinical data in the future, we anticipate gaining a deeper understanding of the role of these medications in the management of candidiasis.
